# Dynamic phenotypic correlates of social status and mating effort in male and female red junglefowl, *Gallus gallus*

**DOI:** 10.1111/jeb.13541

**Published:** 2019-09-28

**Authors:** Rômulo Carleial, Grant C. McDonald, Tommaso Pizzari

**Affiliations:** ^1^ Edward Grey Institute Department of Zoology University of Oxford Oxford UK; ^2^ Department of Ecology University of Veterinary Medicine Budapest Budapest Hungary

**Keywords:** badge of status, comb, condition dependence, cost of mating, sexual coercion, sexual selection, social status

## Abstract

Despite widespread evidence that mating and intrasexual competition are costly, relatively little is known about how these costs dynamically change male and female phenotypes. Here, we test multiple hypotheses addressing this question in replicate flocks of red junglefowl (*Gallus gallus*). First, we test the interrelationships between social status, comb size (a fleshy ornament) and body mass at the onset of a mating trial. While comb size covaried positively with body mass across individuals of both sexes, comb size was positively related to social status in females but not in males. Second, we test for changes within individuals in body mass and comb size throughout the mating trial. Both body mass and comb size declined at the end of a trial in both sexes, suggesting that mating effort and exposure to the opposite sex are generally costly. Males lost more body mass if they (a) were socially subordinate, (b) were chased by other males or (c) mated frequently, indicating that subordinate status and mating are independently costly. Conversely, females lost more body mass if they were exposed to a higher frequency of coerced matings, suggesting costs associated with male sexual harassment and female resistance, although costs of mating *per se* could not be completely ruled out. Neither competitive nor mating interactions predicted comb size change in either sex. Collectively, these results support the notion that sex‐specific costs associated with social status and mating effort result in differential, sex‐specific dynamics of phenotypic change.

## Graphical abstract

We present empirical evidence that social status and mating behaviours are associated with sex‐specific dynamic changes in body mass, but not ornament expression, in replicate groups of polygynandrous red junglefowl (*Gallus gallus*). Photo by Dom Cram.

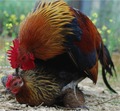

## 1 INTRODUCTION

Sexual reproduction is typically associated with costs of intrasexual competition, mating and mate choice (Foley et al., 2018; Lehtonen, Jennions, & Kokko, 2012). These costs can be substantial and have been indicated as key drivers of phenotypic variation among individuals of both sexes (e.g. Andersson, 1994; Biernaskie, Grafen, & Perry, 2014; Hare & Simmons, 2019). In males, particularly in non‐monogamous species, reproductive costs are largely associated with intrasexual competition over access to mating and fertilization, and with sexual selection, promoting phenotypes that confer a competitive advantage through direct intrasexual competition (e.g. male–male combat) and/or intersexual mechanisms of mate selection (Andersson, 1994; Andersson & Simmons, 2006; Darwin, 1871). Differential costs associated with mechanisms of intrasexual competition can in principle explain variation in male competitive ability. In social species, intrasexual competition is often regulated by social hierarchies (Drews, 1993), with dominant males having privileged access to reproductive opportunities (e.g. Cowlishaw & Dunbar, 1991; Eason & Sherman, 1995; Klinkova, Hodges, Fuhrmann, Jong, & Heistermann, 2005; Majolo, Lehmann, Vizioli, Schino, & Sapienza, 2012; McElligott et al., 2001). However, the differential costs associated with being dominant and subordinate are unclear. Some studies have shown that retaining high status is particularly stressful, while others have indicated that low status is associated with relatively high levels of physiological stress (Abbott et al., 2003; Creel, 2001; Gesquiere et al., 2011; Goymann & Wingfield, 2004; see Habig, Doellman, Woods, Olansen, & Archie, 2018 for a recent meta‐analysis). Similarly, variation in attractiveness to females may be driven by differential costs associated with the expression of male ornaments or courtship (Barske, Schlinger, Wikelski, & Fusani, 2011; Hamilton & Zuk, 1982; Vehrencamp, Bradbury, & Gibson, 1989). Individual variation in sexually selected male phenotypes may thus reflect condition dependence and capture additive genetic variance in the ability of an individual to sustain costly investments (Lorch, Proulx, Rowe, & Day, 2003; Rowe & Houle, 1996; Tomkins, Radwan, Kotiaho, & Tregenza, 2004). For example, recent experimental studies in invertebrates suggest that the costs of maintaining sexually selected weaponry are felt differentially across individuals and phenotypes and are subject to dynamic changes (Joseph, Emberts, Sasson, & Miller, 2018; Somjee, Woods, Duell, & Miller, 2018). In females on the other hand, phenotypic variation is thought to reflect mainly differential investment in fecundity and the energetic requirements associated with mating, and mate discrimination (Bakker, Künzler, & Mazzi, 1999; Clutton‐Brock, 2009; Qvarnström & Forsgren, 1998; Wong & Candolin, 2005). Evidence that these female behaviours incur immediate costs, however, remains ambiguous. For example, female resistance to male mating advances (sexual harassment) is thought to be energetically costly (Jormalainen, Merilaita, & Riihimaki, 2001; Perry, Sharpe, & Rowe, 2009), although measurements of these energetic costs are often indirect (reviewed in Fox, Head, & Jennions, 2019). Establishing how male and female phenotypes change within and among individuals in relation to intrasexual competition and mating effort is therefore important to understand mate preferences, the maintenance of variation in sexually selected traits, and sexual conflict (Andersson & Simmons, 2006; Arnqvist & Rowe, 2005; Dale, Dey, Delhey, Kempenaers, & Valcu, 2015; Foley et al., 2018; Joseph et al., 2018; Kokko, Brooks, Jennions, & Morley, 2003; Sánchez‐Tójar, Nakagawa, et al., 2018; Somjee et al., 2018).

Here, we characterize male and female patterns of interindividual variation and dynamic within‐individual changes associated with intrasexual competition and mating interactions in replicate groups of red junglefowl (*Gallus gallus*). In the wild, this sexually dimorphic species forms polygynandrous social groups, characterized by sex‐specific social hierarchies (Collias & Collias, 1967, 1996; Collias, Collias, Hunsaker, & Minning, 1966). Captive populations of red junglefowl and populations of the related domestic fowl (*G. domesticus*) have been extensively studied in sexual selection research (e.g. Parker & Ligon, 2002; Pizzari & McDonald, 2019; Zuk, Thornhill, Ligon, & Johnson, 1990). Unless populations are heavily female‐biased, matings are often initiated by males, and females tend to resist the majority of these attempts, leading to mating struggles and sexual coercion (reviewed in Pizzari & McDonald, 2019). While such mating effort appears to involve considerable energetic cost for both males and females, little is known about the association between mating effort and phenotypic variation.

Socially dominant, more aggressive males are typically favoured in sexual selection, through both intrasexual and intersexual mechanisms (Pizzari & McDonald, 2019). There is some evidence that male body mass is positively related to the outcome of dyadic competitive contests, suggesting that achieving high social status might require condition (Ligon, Thornhill, Zuk, & Johnson, 1990). It is however unclear whether attaining high social status incurs more costs than remaining at the bottom of the hierarchy. On the one hand, low‐ranking males may suffer from limited access to food and other resources; on the other hand, high‐ranking males may face higher energetic costs to maintain dominant status via aggressive interactions. Dominant males also invest less time feeding and resting than subordinate males (Pizzari & McDonald, 2019), suggesting that over time, high‐ranking males may be prone to lose weight. In addition, several studies have shown a female preference to mate with males sporting a large comb (a fleshy head ornament; reviewed in Parker & Ligon, 2002; Pizzari & McDonald, 2019). Comb expression is characterized by high phenotypic plasticity, and the significance of male comb size variation has received considerable focus. Increasing evidence indicates that comb size captures condition through sensitivity to immune challenges (reviewed in Parker & Ligon, 2007; Pizzari & McDonald, 2019). First, comb size is dependent on testosterone plasma levels (Allee, Collias, & Lutherman, 1939; Hardesty, 1931), which may depress the ability of an organism to respond to immune challenges (Folstad & Karter, 1992). Consistent with this idea, male comb size responds to parasitic infections (Zuk, Thornhill, Ligon, & Johnson, 1990; but see Chappell, Zuk, Johnsen, & Kwan, 1997) and domestic chicken lines artificially selected for strong immunological responses tend to display both low plasma levels of testosterone and small combs (Verhulst, Dieleman, & Parmentier, 1999). Second, comb size may also reflect the effect of free radicals released by phagocytic activation triggered by parasitic infections (von Schantz, Bensch, Grahn, Hasselquist, & Wittzell, 1999). This has led researchers to suggest that comb size can function as an honest signal of individual quality and particularly of immunocompetence (Zuk & Johnsen, 1998, 2000; Zuk, Johnsen, & Maclarty, 1995; Zuk, Thornhill, Ligon, & Johnson, 1990). Additionally, testosterone control of male comb size has led to the suggestion that comb size may function as a badge of status (Ligon et al., 1990). Such badges are expected to evolve to signal individual quality and competitive ability, allowing individuals to minimize the risks of physical contests based on their predicted outcome (Sánchez‐Tójar, Nakagawa, et al., 2018; Santos, Scheck, & Nakagawa, 2011). Consistent with this idea, male comb size appears to be sensitive to social challenges. For example, subordinate males experience comb shrinkage in the presence of dominant males (Cornwallis & Birkhead, 2008; Zuk & Johnsen, 2000), and individuals isolated from dominance interactions experience faster comb growth than individuals that remain in flocks (Parker, Knapp, & Rosenfield, 2002). Comb size was also positively correlated with social status among males in some studies (Graves, Hable, & Jenkins, 1985; Johnsen, Zuk, & Fessler, 2001; Ligon et al., 1990; Parker et al., 2002; Zuk, Johnson, Thornhill, Ligon, & David, 1990; Zuk, Thornhill, Ligon, Johnson, et al., 1990; reviewed in Pizzari & McDonald, 2019). Evidence for a relationship between male comb size and social status, however, remains inconsistent. For example, Johnsen et al. (2001) found that comb size and, to a lesser extent, body size and body mass were associated with male social status in the first year of their study, but failed to find an association in the following year. Similarly, Ligon et al. (1990) found that comb size predicted the outcome of competitive dyadic contests between yearling red junglefowl males that had been socially isolated, but not the outcome of dyadic contests between socially familiar yearling and older males. More recently, comprehensive studies have produced inconsistent evidence for badges of status in other avian systems. While some studies have provided evidence consistent with badges of status reliably signalling individual quality in both sexes (e.g. López‐Idiáquez, Vergara, Fargallo, & Martínez‐Padilla, 2016; Santos et al., 2011), others have failed to support this hypothesis (e.g. Sánchez‐Tójar, Nakagawa, et al., 2018).

As in males, social status modulates access to resources in females in both red junglefowl and domestic fowl populations (Collias, Collias, & Jennrich, 1994; Sanctuary, 1932; Shimmura et al., 2008), and evidence suggests that female comb size is associated with female social status in both taxa (Bradshaw, 1992; Collias, 1943; Forkman & Haskell, 2004; Guhl & Ortman, 1953; Martin, Beaugrand, & Lague, 1997), although body mass may also play a role (Cloutier & Newberry, 2000; Zuk, Kim, Robinson, & Johnsen, 1998). Moreover, as in males, female comb size correlates with plasma levels of steroid hormones, which in turn are good predictors of female aggressiveness (Allee et al., 1939; Collias, 1943). Large‐combed females usually initiate and win dominance contests or are promptly avoided by other females, suggesting that large combs are associated with a competitive advantage and might be used for social signalling and individual recognition (Guhl & Ortman, 1953). Additionally, female comb size has been shown to be positively correlated with reproductive investment (Cornwallis & Birkhead, 2007; Pizzari, Cornwallis, Løvlie, Jakobsson, & Birkhead, 2003; Wright et al., 2008), and dominant males may use female comb size as a cue during mating decisions (Cornwallis & Birkhead, 2007; Pizzari et al., 2003).

Here, we studied replicate mating groups of red junglefowl to characterize variation in traits associated with intrasexual competition and mating interactions. We first characterize variation among individuals of each sex and then investigate dynamic changes within individual males and females over time.

## 2 MATERIALS AND METHODS

### 2.1 Behavioural observations

**Figure 1:**
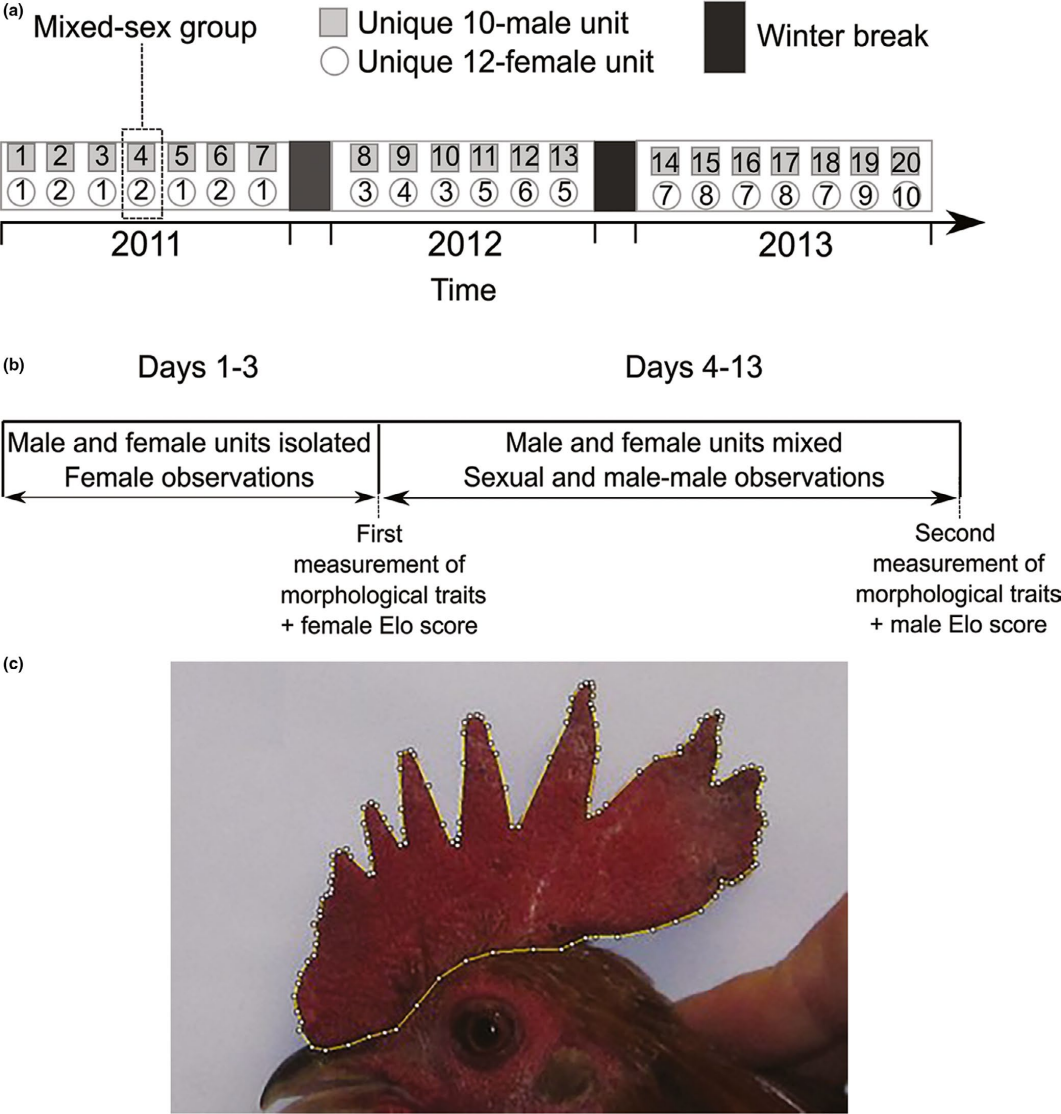
Schematics of (a) the experimental design outlining how the 20 replicate experimental mating groups of red junglefowl (*Gallus gallus*) were assembled from unique sets of female and male groups over the course of 3 years, (b) the timeline of the observation trial of each mating group, and (c) comb area measurement, here highlighted in an individual male

Behavioural observations were performed by a single observer (GCM) in a captive population of red junglefowl at the University of Oxford field station in Wytham, UK. We observed 20 mixed‐sex groups (12 females and 10 males per group) in an outdoor arena (5.8 × 16.5 m) over three breeding seasons (April–October, 2011–2013). The relatively small group size, its social cohesiveness and the size of the outdoor arena allowed for observation of the whole group at all times during the trial. Birds used in the study were sexually mature adults ranging from 1 to 7 years old. Each group was exposed to a 13‐day trial. In the first three days of a trial, males and females were housed in sex‐specific pens in order to allow social familiarization with the other members of the same sex and the formation of sex‐specific hierarchies. We then assembled the mixed‐sex mating group by placing these male and female groups together in the same experimental pen on the morning of the fourth day (Figure 1a) and conducted behavioural observations for 10 consecutive days (Figure 1b), from 5am to 9am and from 6 pm to 9 pm (see McDonald, Spurgin, Fairfield, Richardson, & Pizzari, 2017 for details). In total, 127 unique males and 78 unique females were used in this study, with 61 males and 48 females being reused across groups due to limitation of new unique birds (see Table S1 for a summary). In particular, the limited number of females available for the study meant that we assembled 10 unique female groups, which were used multiple times with different male groups to form 20 unique mixed‐sex mating groups (Figure 1). On six occasions, a female became sick or died during a trial and was replaced immediately with a new female to maintain a constant sex‐ratio among groups. Replacement females were randomly selected using the ‘sample’ function in R to select the identity of an individual from the pool of available reproductively mature females. All agonistic interactions between males (pecks, fights, waltzes, chases and avoidances) were recorded during the 10 days of mixed‐sex observations and were posteriorly used to establish individual social status (see below). We considered the loser of these interactions the male that retreated at least one body length from the other male (Froman, Pizzari, Feltmann, Castillo‐Juarez, & Birkhead, 2002; Johnsen et al., 2001; Wilson, Nelson, & Evans, 2009). When it was not obvious which individual had won the interaction, the outcome was defined as a draw. Agonistic interactions between females were recorded during three days immediately before the 10 days of mixed‐sex observations (i.e. days 1–3), when females were still isolated from males. Observations lasted for 3 h between 11am and 6 pm. We found this to be necessary given the more subtle nature of female behaviour, which are harder to detect when females interact with males. Male courtship (i.e. waltzing to a female), copulation attempts and successful copulations (i.e. when we observed direct cloacal contact or the male tail bending towards the female cloaca) were also recorded during the 10 days of mixed‐sex observations (i.e. day 4–13). Additionally, we used published methods to score the level of female resistance during copulation attempts, which ranged from 1 (female solicitation) to 6 (female was chased by the male, grabbed and sexually coerced; Løvlie, Cornwallis, & Pizzari, 2005; Løvlie & Pizzari, 2007). Starting on the second day of the trial, eggs were collected each morning up to, and including, one day post‐trial. Eggs were assigned to females using parentage analyses that are described elsewhere (McDonald et al., 2017). In short, eggs were incubated artificially for at least five days, and embryo tissue samples were then collected and stored in absolute ethanol at 4°C prior to parentage assignment. We disregarded any egg for which parentage was not assigned molecularly with at least 95% confidence (see McDonald et al., 2017) and the total number of identifiable eggs per female was used as the measure of her fecundity.

### 2.2 Phenotypic measurements

Body mass and comb size were measured on two occasions for each bird: on the third day of a trial before the male and female groups were assembled together as a single mixed‐sex mating group, and one day post‐trial. On each occasion, birds were weighed to the nearest 10 g with a digital hanging scale, and their combs were photographed using a Canon Ixus 107 with 12.1mb/p resolution, holding the bird at a fixed distance and in the same position in relation to the camera, with a scale attached to the wall behind the bird which allowed comb measurements to be performed digitally. To do so, we drew a polygon around the comb (Figure 1c) using the software ImageJ v1.51k, and the comb area was calculated using the scale in the photo as the reference. The pictures were chosen at random. In order to access the repeatability of comb measurements, a single observer (RC) measured the comb of a random subset of 44 individuals (24 females and 20 males) on two separate occasions (i.e. at least a week apart), and measurements were compared using a repeatability analysis implemented in the R software (R Core Team 2017, version 3.4.2) package ‘rptR’ v0.9.2 (Stoffel, Nakagawa, & Schielzeth, 2017). The different measurements were highly repeatable (*r* = .982, *p* < .001), giving us confidence of detecting biological differences in comb size within and between individuals. Due to methodological constraints, we were unable to get estimates of skeletal body size (e.g. tarsus length), and thus, the first weighting of a bird captures both skeletal size and residual mass for size. Changes in body weight within individual birds, between the first and second weighing on the other hand, are taken to represent changes in residual body mass.

### 2.3 Social status

Social status was estimated using the randomized Elo‐rating method implemented in the R package ‘aniDom’ v.0.1.4 (Farine & Sánchez‐Tójar, 2017; Sánchez‐Tójar, Schroeder, & Farine, 2018). In the original Elo‐rating method (Elo, 1978; Neumann et al., 2011), individuals start out with equal ratings, which are updated after each dyadic agonistic interaction, with winners gaining points and loser losing points. The amount of points gained or lost in each interaction is dependent upon the scores of the two individuals at the moment of the interaction: a higher‐rated individual receives fewer points if it wins against a lower‐rated individual, whereas the latter receives more points if it wins against a higher‐rated individual. The randomized method expands on this by generating replicate data sets, which randomize the order of the dyadic interactions, yielding more robust estimates of Elo scores (Sánchez‐Tójar, Schroeder, et al., 2018). We assessed the repeatability of 1,000 replicates for each of the 20 male groups, and the individual's mean cumulative Elo score (i.e. the score held at the last day of the trial) calculated from these randomizations was used as the measurement of social status. Because draws were rare (1.18% of males’ agonistic interactions), they were removed from calculations of Elo scores, following McDonald and Shizuka (2012). We followed a similar protocol with females; however, in this case, we calculated mean cumulative Elo scores using randomizations of the dyadic agonistic interactions that took place during the first three days when the female group was isolated from the males. No draws were recorded among females, so all agonistic interactions were used to calculate Elo scores. Additionally, we calculated the transitivity (i.e. linearity) of the dominance hierarchies of each sex using the triangle transitivity method (t_tri_, *sensu*; Shizuka & McDonald, 2012) and the stability (i.e. how often individuals swap ranks) of these hierarchies using the modified stability index (S_t_, Neumann et al., 2011; McDonald & Shizuka, 2012), both of which are implemented in the R package ‘EloRating’ v0.46.8 (Neumann et al., 2011).

On average, we recorded 231.69 (*SD* = 141.97) agonistic interactions per individual male over the 10 days of a trial, and 41.27 (*SD* = 28.04) agonistic interactions per female over the three days of observation preceding the trial, that is prior to the introduction of males. These numbers of interactions are well above the sampling effort recommended by a previous study (i.e. *n *= 20; Sánchez‐Tójar, Schroeder, et al., 2018), suggesting that hierarchies were reliably estimated. The mean repeatability of randomized Elo scores was 0.93 (*SD* = 0.06) in males and 0.95 (*SD* = 0.04) in females. Male dominance hierarchies (Figure S1) were relatively stable (${\bar{S}}_{t}$ = 0.91; *SD* = 0.06) and linear (${\bar{t}}_{\mathrm{tri}}$ = 0.79, *SD* = 0.16). Female dominance hierarchies (Figure S2) were on average slightly less stable (${\bar{S}}_{t}$ = 0.83; *SD* = 0.09) and more linear (${\bar{t}}_{\mathrm{tri}}$ = 0.86, *SD* = 0.16) than male dominance hierarchies.

### 2.4 Statistical analyses

We used linear mixed‐effects models implemented in the R packages ‘lme4’ 1.1‐21 (Bates, Mächler, Bolker, & Walker, 2015) and ‘lmerTest’ 3.1‐0 (Kuznetsova, Brockhoff, & Christensen, 2017). All models included individual identity, female group identity (i.e. the identity of the unique group of 12 females used in each trial, Figure 1a) and mixed‐sex mating group identity as random effects to control for the pseudo‐replication arising from the use of repeated individuals and to allow the intercept of each group to vary. Model residuals were explored for any violation of assumptions (e.g. heteroscedasticity and non‐normality) and collinearities among covariates were tested with the variance inflation factor (VIF) method implemented in the package ‘car’ v3.0‐3 (Fox & Weisberg, 2019). We followed Zuur, Leno, and Elphick (2010) by considering any VIF ≥ 3 as a sign of collinearity, in which case the covariate with higher VIF was removed. *p* values and denominator degrees of freedom for fixed effects were calculated using *F* tests with Satterthwaite's approximation. In presenting statistical inference, we attempt to follow recent recommendations (Amrhein, Greenland, & Mcshane, 2019) within the journal's format.

#### 2.4.1 Interindividual relationships between status, comb size and body mass

We first characterized among individual males and among individual females, the relationship between body mass and comb size, between body mass and social status, and between social status and comb size, and female traits. We investigated whether body mass or comb size were related to social status by modelling the individual Elo score as the response variable and body mass and comb area as explanatory variables. The individual's age (in years) was modelled as a covariate in these models to control for any confounding effect age may have on social status (e.g. via greater social experience). For females, we also modelled fecundity as a covariate, since previous work indicates that measures of female fecundity in fowl populations are correlated with female social status (Collias et al., 1994; Sanctuary, 1932) and comb size (Cornwallis & Birkhead, 2007; Pizzari et al., 2003; Wright et al., 2008). The inclusion of female fecundity as a covariate controlled for any potential confounding effect of fecundity on status and comb size variation. Finally, there is evidence that relationships between female comb size and other phenotypic traits tend to show patterns of non‐linearity (Pizzari et al., 2003; Wright et al., 2008), so we explored this possibility by including a quadratic component in the model.

#### 2.4.2 Intraindividual temporal changes

We tested whether body mass and comb size showed consistent changes within individual males and within individual females across time. We compared the first measurement, taken the day before birds were moved to the experimental mixed‐sex mating group, with the second measurement, taken on the day after the trials were complete. Body mass or comb size was used as the response variable and treatment (before/after) as the independent variable. For logistical reasons (i.e. single observer), trials were run sequentially within a breeding season, leading to variation in the time of year each trial was conducted, for example early *versus* late in the breeding season (variation in seasonality). To exclude the possibility of variation in our data being driven by such seasonality alone, we investigated whether changes in body mass or comb size varied consistently over the breeding season. To do so, the first day of the earliest trial across the three years of study was set as 0 and later groups were numbered according to the number of days elapsed from day 0 to the day that trial began. This continuous variable therefore indicates the stage of the breeding season in which each group was initiated and was then used as a proxy of seasonality. Changes in phenotype were calculated by subtracting the measurement of initial body mass or comb size from the second measurement. Following Kelly and Price (2005), we calculated the amount of change between two measurements that is expected solely due to the regression to the mean effect (i.e. when due to measurement error, individuals that are far away from the mean tend to approximate the mean in a posterior measurement), and discounted this amount from the original differences of phenotypic measurements. We only used individuals for which we had information on both phenotypic measurements (i.e. initial and final comb size or body mass); for example, birds that were swapped during the mating trial or that were incorrectly measured were not included (see sample sizes in table results).

We investigated whether the rate of phenotypic change within males and females were dependent on a bird's social status. We used the mean cumulative Elo score (i.e. across all randomizations) held by the individual following his last interaction over the course of the trials (3 days in the case of females and 10 days in the case of males) as the measure of social status. This cumulative value was modelled as the independent variable and used to predict changes in body mass and comb sizes in both sexes. Because we had information on male hierarchies over the course of the 10‐day mating trial (as opposed to only 3 days for females), we tested whether the stability of a male's position in the dominance hierarchy was related to consistent changes in body mass and comb size. We did this in two ways. First, stability was represented by the number of days, over the 10‐day mating trial, in which a male changed social rank (i.e. ended the day in a different rank than he had started). Stability ranged from 0, if the male never changed rank, to 9, for a male that finished each day in a different rank. Therefore, regardless of how many positions the male climbed or lost in the hierarchy during a single day, we regarded it as a single change (i.e. going from 2nd to 9th or 6th to 7th in the hierarchy were both expressed as one change), with these discrete ranks being derived from daily measurements of Elo scores. We hypothesized that frequent changes in rank would be stressful to males and have a negative impact in their body mass and comb size. However, changes in the social rank of a focal male might also arise as indirect consequence of changes in the rank of other males, without the focal male being necessarily involved in agonistic interactions (see Strauss & Holekamp, 2019, for similar argument about ‘passive’ processes). To avoid the potentially confounding effect of these indirect processes and capture the potential costs of agonistic interactions associated with changes in social rank, we also considered the total number of agonistic interactions of a focal male as an alternative proxy of social stress. We hypothesized that a higher number of interactions would reflect a higher cost for the focal male. Additionally, we investigated whether the number of times a male chased a rival, or was chased by him, impacted his body mass and comb size.

Finally, we investigated potential costs of mating effort in driving dynamic changes in the expression of male and female phenotypes. For mating behaviour, we tested whether courtship (i.e. number of waltzes performed by a male or received by a female), mating success (i.e. number of unique copulation partners) and the total number of copulation attempts (both successful and unsuccessful) are associated with changes in body mass or comb size in either sex. We hypothesized that the energetic investment by more sexually active individuals would lead to losses in body mass and reduction in comb size. Additionally, we tested whether the number of coerced copulation attempts suffered by females (scores from 4 to 6 as described in Løvlie et al., 2005) are associated with changes in female body mass and comb size. Coerced copulations represent a subset of the total number of copulation attempts. We predicted the stress of harassment by males would lead to a decrease in body mass and comb size and that females that were exposed to higher levels of sexual harassment would lose more body mass and suffer a greater reduction in comb size. We also investigated whether a female's fecundity (number of eggs laid during a trial) was associated with changes in her body mass and comb size. Given that more fecund females tend to be more sexually active (e.g. Løvlie & Pizzari, 2007; McDonald, Spurgin, Fairfield, Richardson, & Pizzari, in press), we predicted that higher fecundity would expose females to more intense sexual harassment, leading to greater loss in body mass and reduction in comb size.

## 3 RESULTS

### 3.1 Interindividual relationships between status, comb size and body mass

**Figure 2:**
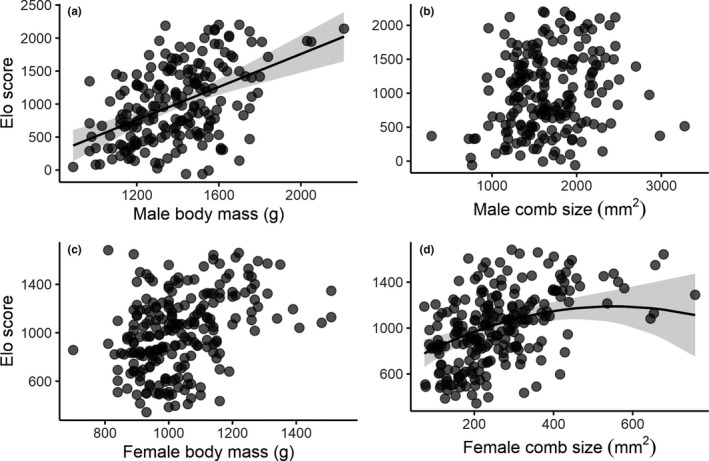
Relationship between social status and two condition dependent sexual traits in male and female red junglefowl (*Gallus gallus*). Relationship between Elo scores (i.e., social status) and (a) male body mass, (b) male comb size, (c) female body mass and (d) female comb size. Data points represent individual birds, with darker regions indicating data point overlaps. Shaded areas around the regression line represent the 95% confidence intervals

Body mass and comb size were positively intercorrelated in both males (LMM: *F*_1,192.7_ = 29.23, *p *< .001, estimate ± *SE*: 0.68 ± 0.13) and females (LMM: *F*_1,208.6_ = 21.86, *p *< .001, estimate ± *SE*: 0.21 ± 0.04), so both variables were entered as covariates when we analysed their relationship with social status. We found that across groups, when age was controlled for, there was a statistically significant and positive association between body mass and social status in males (LMM: *F*_1,133.5_ = 29.94, *p *< .001, estimate ± *SE*: 1.25 ± 0.23, Table 1, Figure 2a), but not in females (LMM: *F*_1,187.4_ = 0.02, *p *= .896, estimate ± *SE*: 0.02 ± 0.18, Table 1, Figure 2c). Conversely, there was no statistically significant association between comb size and status in males (LMM: *F*_1,124.7_ = 0.02, *p *= .901, estimate ± *SE*: 0.01 ± 0.10, Table 1, Figure 2b), but in females, there was a statistically significant and positive linear relationship between comb size and social status (LMM: *F*_1,143.3_ = 10.23, *p *= .002, estimate ± *SE*: 1.98 ± 0.62, Table 1), and evidence for a statistically weaker negative quadratic relationship (LMM: *F*_1,137.3_ = 3.98, *p *= .048, estimate ± *SE*: −0.002 ± <0.001, Tabe 1, Figure 2d). Despite these overall patterns, there was considerable variation among groups in the relationships between comb size and status, and between body mass and status (Figure S3).

**Table 1:** Linear mixed‐effect models (LMMs) results for the relationship between multiple traits and social status in male and female red junglefowl (*Gallus gallus*)

	Estimate	*SE*	*F*	ddf	*p*
Male Elo score					
Fixed effects					
Intercept	−756.7	251.8	–	137.13	–
Age	−1.8	23.33	0.01	118.09	.939
Body mass	1.25	0.23	29.94	133.5	**<.001**
Comb size	0.01	0.1	0.02	124.72	.901
Random effects					
σ^2^	227123.3				
τ_00 Male identity_	35531.5				
τ_00 Group identity_	1.12^−14^				
τ_00 Female group identity_	2.37^−15^				
Observations	200				
Female Elo score					
Fixed effects					
Intercept	334.84	183.4	–	*** ***167.8	–
Age	90.55	15.88	32.52	79.61	**.002**
Body mass	0.02	0.18	0.02	187.41	.897
Comb size	1.98	0.62	10.23	143.29	**<.001**
Comb size^2^	−0.002	<0.001	3.98	137.3	**.048**
Fecundity	15.54	6.39	5.89	207.92	**.016**
Random effects					
σ^2^	22754.62				
τ_00 Female identity_	35872.78				
τ_00 Group identity_	2.82^−3^				
τ_00 Female group identity_	1438.73				
Observations	226				

Note

*p* values of fixed effects are based on F tests with Satterthwaite's approximation and are highlighted in bold when results are statistically significant (*p *< .05).

Abbreviations: *SE*, standard error; *F*,* F* statistics; ddf, denominator degrees of freedom; σ^2^, residual variance; τ00, random intercept variance.

### 3.2 Intraindividual temporal changes

We found a statistically significant decrease in body mass (males: LMM: *F*_1,245.5_ = 103.78, *p *< .001, estimate ± *SE*: 60.1 ± 5.9; females: LMM: *F*_1,376.7_ = 83.15, *p *< .001, estimate ± *SE*: 39.41 ± 4.32) and comb size (males: LMM: *F*_1,249.4_ = 7.99, *p *= .005, estimate ± *SE*: 28.5 ± 10.1; females: LMM: *F*_1,378.6_ = 36.69, *p *< .001, estimate ± *SE*: 14.6 ± 2.41) in both sexes during the 10 days of a trial in which males and females mixed and interacted freely. We found no evidence that the decline in body mass varied consistently over the breeding season in neither males (LMM: *F*_1,17_ = 2.90, *p *= .107, estimate ± *SE*: 0.25 ± 0.15, Figure S4a) or females (LMM: *F*_1,16.1_ = 1.72, *p *= .209, estimate ± *SE*: −0.19 ± 0.15, Figure S4c). Similarly, there was no evidence that the decline in female comb size was consistently affected by seasonality (LMM: *F*_1,17_ = 2.94, *p *= .105, estimate ± *SE*: −0.14 ± 0.08, Figure S4d). However, there was a statistically significant tendency for the male comb to shrink more as the breeding season progressed (LMM: *F*_1,18.1_ = 6.02, *p *= .025, estimate ± *SE*: −0.73 ± 0.29, Figure S4b). Accounting for this effect in the following analyses did not affect qualitatively our results, so we do not report it here.

**Figure 3:**
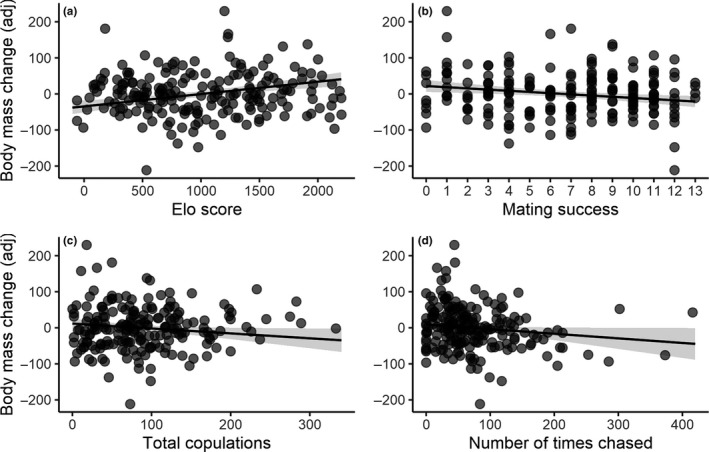
Relationship between changes in body mass and multiple sexually selected behaviors in male red junglefowl (*Gallus gallus*). Adjusted body mass difference was calculated by subtracting the individual's body mass (g) on the day following the mixed‐sex trial from its body mass on the day before the trial, while simultaneously correcting for the regression to the mean effect. Therefore, the adjusted body mass difference does not represent the actual differences in body mass. Adjusted changes in body mass were associated to (a) male social status, (b) male mating success (i.e., number of unique copulation partners), (c) total number of copulations, and (d) the number of times that a male was chased by other males. The removal of outliers does not change qualitatively the results. Data points represent individual males, with darker regions indicating data point overlaps. Shaded areas around the regression line represent the 95% confidence intervals

After controlling for any regression to the mean, males that were initially heavier lost more body mass than initially lighter males (LMM: *F*_1,134.1_ = 35.42, *p *< .001, estimate ± *SE*: −0.11 ± 0.02, Table 2), and large‐combed males suffered a larger degree of comb shrinking than males with initially smaller combs (LMM: *F*_1,185.4_ = 4.64, *p *= .033, estimate ± *SE*: −0.03 ± 0.01, Table 2), and these relationships were independent of social status. We therefore entered initial body mass or comb size as a covariate in all statistical models in which body mass change or comb size change (respectively) were used as the response variable. Controlling for initial body mass, we found a statistically significant and positive association between social status and body mass change, such that males of higher status either gained or tended to lose less mass over the course of the 10‐day trial (LMM: *F*_1,187.3_ = 21.52, *p *< .001, estimate ± *SE*: 0.03 ± <0.01, Table 2, Figure 3a). On average, each standard deviation (*SD*) increment in Elo score resulted in a 19.7 g increment in body mass. We found that neither the number of times a male swapped rank (LMM: *F*_1,192_ = 1.02, *p *= .314, estimate ± *SE*: 1.47 ± 1.45, Table 2) nor the total number of agonistic interactions he experienced (LMM: *F*_1,94.7_ = 2.84, *p *= .095, estimate ± *SE*: −0.05 ± 0.03) were statistically associated with changes in his body mass. We found a statistically borderline nonsignificant trend for males that mated with more females to have lost more body mass (LMM: *F*_1,170_ = 3.67, *p *= .057, estimate ± *SE*: −2.13 ± 1.11), whereas there was no statistically significant association between the total number of copulation attempts and body mass change in males (LMM: *F*_1,154.8_ = 1.17, *p *= .282, estimate ± *SE*: −0.07 ± 0.06). However, when the positive effect of social status was controlled for, we found a statistically significant association between an increased loss in body mass and the number of females mated (i.e. mating success, LMM: *F*_1,177.8_ = 9.40, *p *= .003, estimate ± *SE*: −3.31 ± 1.08, Table 2, Figure 3b), or the total number of copulation attempts (LMM: *F*_1,138_ = 4.58, *p *= .034, estimate ± *SE*: −0.14 ± 0.06, Table 2, Figure 3c). One *SD* increment in mating success translated into 11.9 g of body mass loss on average, while one *SD* increment in copulation attempts translated into 8.47 g of body mass loss. There was no statistically significant association between the number of times a male courted a female (i.e. number of waltzes) and changes in his body mass (LMM: *F*_1,146.3_ < 0.01, *p *= .972, estimate ± *SE*: <0.01 ± 0.06), although there was a statistically weak relationship between these variables when social status was controlled for (LMM: *F*_1,155.6_ = 3.22, *p *= .075, estimate ± *SE*: −0.11 ± 0.06, Table 2). Males that chased other males more often tended to lose less or gain more body mass (LMM: *F*_1,195.4_ = 5.26, *p *= .023, estimate ± *SE*: 0.10 ± 0.04); however, when the confounding effect of social status was controlled for, this relationship was no longer statistically significant (LMM: *F*_1,179.4_ = 0.76, *p *= .384, estimate ± *SE*: −0.05 ± 0.05, Table 2). On the other hand, males that were chased more often by other males tended to lose more body mass (LMM: *F*_1,172.5_ = 13.26, *p *= .001, estimate ± *SE*: −0.22 ± 0.06), and evidence for this relationship, albeit statistically weaker, remained after controlling for status (LMM: *F*_1,136.2_ = 4.02, *p *= .047, estimate ± *SE*: −0.13 ± 0.06, Table 2, Figure 3d). One *SD* in the number of chasing episodes translated on average into 8.22 g lost in body mass. While male body mass changes were related to social status, aggression and some aspects of mating effort, we found little evidence for similar patterns in changes in male comb size. There was no statistically significant association between male comb size change and social status (LMM: *F*_1,177.4_ = 2.58, *p *= .110, estimate ± *SE*: 0.02 ± 0.01, Table 2), chasing other male rivals (LMM: *F*_1,186.1_ = 2.79, *p *= .097, estimate ± *SE*: 0.11 ± 0.06, Table 2), or being chased by them (LMM: *F*_1,195.4_ = 1.55, *p *= .215, estimate ± *SE*: −0.13 ± 0.10, Table 2). Similarly, there was no evidence for a statistically significant relationship between comb size changes and changes in social rank (LMM: *F*_1,195.9_ = 0.04, *p *= .834, estimate ± *SE*: 0.54 ± 2.57, Table 2) or the total number of agonistic interactions (LMM: *F*_1,194.6_ = 0.81, *p *= .369, estimate ± *SE*: 0.04 ± 0.05). Finally, we did not find any statistically significant association between comb size change and any of the measures of male mating effort (LMMs: mating success: *F*_1,194.3_ = 0.71, *p *= .399, estimate ± *SE*: −1.52 ± 1.80; copulation attempts: *F*_1,196_ = 0.06, *p *= .803, estimate ± *SE*: 0.03 ± 0.11; courtship: *F*_1,189.8_ = 0.29, *p *= .591, estimate ± *SE*: 0.05 ± 0.09, Table 2).

**Table 2:** Linear mixed‐effect models (LMMs) results for the relationship between social status and multiple behaviours on changes in body mass and comb size in male red junglefowl (*Gallus gallus*)

Full model	*n*	Fixed effects	Estimates	*SE*	*F*	ddf	*p*	σ^2^	τ00
BMc ~ BM	200	Intercept	152.17	25.88	–	140.34	–	2813	271.5
		Body mass	−0.11	0.02	35.42	134.1	**<.001**		
BMc ~ BM* + Elo	200	Intercept	183.92	25.75	–	143.54	–	2388.63	430.41
		Body mass	−0.16	0.02	60.56	153.22	**.015**		
		Elo score	0.03	<0.01	21.52	187.27	**<.001**		
BMc ~ BM* + Elo* + C^a^	200	Intercept	184.51	25.78	–	142.83	–	2389.87	433.71
		Body mass	−0.16	0.02	61.43	154.11	**<.001**		
		Elo score	0.04	0.01	16.63	192.91	**<.001**		
		Chase (actor)	−0.05	0.05	0.76	179.39	.384		
BMc ~ BM* + Elo* + C^r^	200	Intercept	190.65	25.8	–	147.2	–	2332.74	446.75
		Body mass	−0.15	0.02	54.52	154.84	**<.001**		
		Elo score	0.03	0.01	11.87	195.72	**.001**		
		Chase (receiver)	−0.13	0.06	4.02	136.21	**.047**		
BMc ~ BM* + Elo* + TC	200	Intercept	211.88	28.27	–	165.18	–	2283	497.62
		Body mass	−0.17	0.02	67.18	166.25	**<.001**		
		Elo score	0.04	0.01	25.43	182.91	**<.001**		
		Total copulations	−0.14	0.06	4.58	138	**.034**		
BMc ~ BM* + Elo* + Co	200	Intercept	190.79	25.96	–	150.83	–	2309.93	485.64
		Body mass	−0.16	0.02	62.07	157.04	**<.001**		
		Elo score	0.04	0.01	25.2	185.94	**<.001**		
		Courtship	−0.11	0.06	3.22	155.59	.075		
BMc ~ BM* + Elo* + MS	200	Intercept	213.85	26.99	–	157.25	–	2197.98	526.78
		Body mass	−0.17	0.02	68.13	159.91	**<.001**		
		Elo score	0.04	0.01	27.93	189.07	**<.001**		
		Mating success	−3.31	1.08	9.4	177.77	**.003**		
BMc ~ BM* + Elo* + Rs	200	Intercept	176.76	26.92	–	153.31	–	2384.91	435.37
		Body mass	−0.16	0.02	59.61	154.69	**<.001**		
		Elo score	0.04	0.01	21.79	194.71	**<.001**		
		Rank stability	1.47	1.45	1.02	192.04	.314		
CSc ~ CS	199	Intercept	49.73	25.55	* *	171.23		7002	1804.56
		Comb size	−0.03	0.01	4.64	185.39	**.033**		
CSc ~ CS* + Elo	199	Intercept	41.03	26.06	–	130.77	–	6934	1834.33
		Comb size	−0.04	0.02	6.06	184.6	**.015**		
		Elo score	0.02	0.01	2.58	177.43	.11		
CSc ~ CS* + C^a^	199	Intercept	44.03	25.73	–	170.52	–	6912.3	1884.1
		Comb size	−0.03	0.01	5.09	184.33	**.025**		
		Chase (actor)	0.11	0.06	2.79	186.06	.097		
CSc ~ CS* + C^r^	199	Intercept	53.26	25.79	–	120.8	–	6995.86	1770.31
		Comb size	−0.03	0.01	3.47	186.98	.064		
		Chase (receiver)	−0.13	0.1	1.55	195.4	.215		
CSc ~ CS* + TC	199	Intercept	47.46	27.2	–	178.11	–	7033.11	1821.46
		Comb size	−0.03	0.01	4.64	184.62	**.033**		
		Total copulations	0.03	0.11	0.06	195.99	.803		
CSc ~ CS* + Co	199	Intercept	47.47	25.93	–	173.57	–	7028.52	1806.92
		Comb size	−0.03	0.01	4.88	185.57	**.028**		
		Courtship	0.05	0.09	0.29	189.82	.591		
CSc ~ CS* + MS	199	Intercept	57.33	27.1	–	179.82	–	7021.44	1775.63
		Comb size	−0.03	0.01	4.09	185.8	**.045**		
		Mating success	−1.52	1.8	0.71	194.28	.399		
CSc ~ CS* + Rs	199	Intercept	47.54	27.66	–	176.73	–	7036.48	1812.14
		Comb size	−0.03	0.01	4.61	184.5	**.033**		
		Rank stability	0.54	2.57	0.04	195.91	.834		

Note

*p* values of fixed effects are based on F tests with Satterthwaite's approximation and are highlighted in bold when results are statistically significant (*p *< .05). For more detailed information on predictors, see methods.

Abbreviations: *, covariates; BM, body mass; BMc, body mass change; C^a^, number of chases (actor); C^r^, number of chases (receiver); Co, courtship (i.e. number of waltzes); CS, comb size; CSc, comb size change; Elo, Elo score; MS, mating success; Rs, rank stability; TC, total copulations; n, sample size; *SE*, standard error; F, F statistics; ddf, denominator degrees of freedom; σ^2^, residual variance of the random effects; τ00, sum of the random intercept variances of male identity, female group identity and group identity.

**Figure 4:**
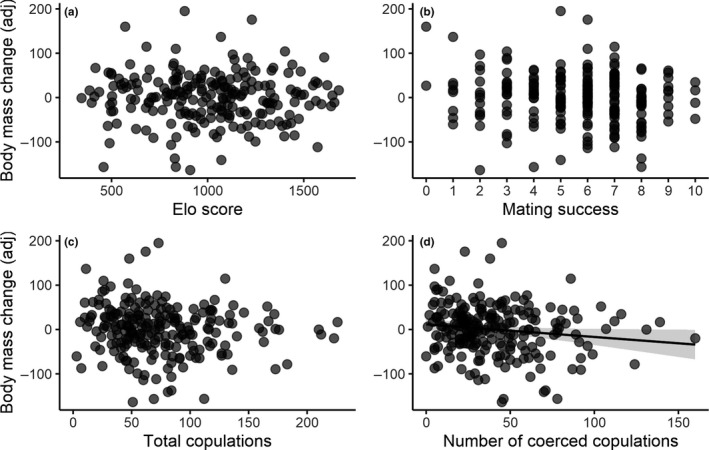
Relationship between changes in body mass and multiple sexually selected behaviors in female red junglefowl (*Gallus gallus*). The y‐axes represent adjusted changes in body mass (see Figure 2). Relationship between the adjusted change in female body mass and (a) female social status, (b) female mating success (i.e., number of unique copulation partners), (c) the total number of copulations, and (d) the number of coerced copulations experienced by a female. The removal of outliers does not change qualitatively the results. Data points represent individual females, with darker regions indicating data point overlaps. Shaded areas around the regression line represent the 95% confidence intervals

As with males, females with initially larger combs experienced more comb shrinking (LMM: *F*_1,218.4_ = 4.18, *p *= .042, estimate ± *SE*: −0.03 ± 0.01, Table 3), even after accounting for any regression to the mean. Therefore, we entered initial comb size as a covariate in all the statistical models where change in comb size was the response variable. There was no evidence for a statistically significant relationship between female social status and changes in body mass (LMM: *F*_1,198.5_ = 0.10, *p *= .751, estimate ± *SE*: <−0.01 ± <0.01, Table 3, Figure 4a) or changes in comb size (LMM: *F*_1,212.4_ = 0.21, *p *= .644, estimate ± *SE*: <0.01 ± <0.01, Table 3). Similarly, there was no evidence of a statistically significant relationship between female mating success (i.e. polyandry) and changes in body mass (LMM: *F*_1,170.6_ = 2.39, *p *= .124, estimate ± *SE*: −2.76 ± 1.79, Table 3, Figure 4b) or between female mating success and comb size change (LMM: *F*_1,213_ = 1.06, *p *= .305, estimate ± *SE*: −0.81 ± 0.79, Table 3). There was no statistically significant association between the total number of copulation attempts experienced by a female and changes in her body mass (LMM: *F*_1,118.9_ = 1.96, *p *= .164, estimate ± *SE*: −0.13 ± 0.09, Table 3, Figure 4c), and there was only a statistically weak tendency for the comb of more sexually active females to shrink more than the comb of less sexually active females (LMM: *F*_1,207.5_ = 3.24, *p *= .073, estimate ± *SE*: −0.08 ± 0.04). When only coerced copulations (i.e. male sexual harassment) were considered, females exposed to more sexual harassment appeared to lose more body mass (LMM: *F*_1,152.3_ = 4.31, *p *= .039, estimate ± *SE*: −0.29 ± 0.14, Table 3, Figure 4d). One *SD* increment in the number of coerced copulations suffered by a female translated on average into an 8.03 g body mass loss in females. We further attempted to disentangle a potential confounding effect of mating from male harassment by entering the total number of copulation attempts as a covariate. In this new analysis, coerced copulations remain statistically associated, albeit weakly, with changes in body mass (LMM: *F*_1,208.3_ = 3.20, *p *= .075, estimate ± *SE*: −2.73 ± 1.77). However, because male harassment is a subset of total copulation attempts, the collinearity between the total number of copulation attempts and coerced copulations is predictably higher than the threshold we had set *a priori* (VIF = 5.16). Therefore, these results must be interpreted with caution. A similar, albeit statistically weaker, pattern was observed when we considered the effect of coerced copulations on comb size changes (LMM: *F*_1,225.3_ = 3.44, *p *= .065, estimate ± *SE*: −0.11 ± 0.06, Table 3). There was no statistically significant association between the number of courtship events that a female received and changes in body mass (LMM: *F*_1,202.9_ = 1.99, *p *= .159, estimate ± *SE*: −0.12 ± 0.09, Table 3) or comb size (LMM: *F*_1,231.7_ < 0.01, *p *= .982, estimate ± *SE*: <−0.01 ± 0.04, Table 3). Finally, there was no statistically significant relationship between female fecundity and changes in body mass (LMM: *F*_1,211.5_ = 1.32, *p *= .252, estimate ± *SE*: −1.91 ± 1.66, Table 3) or comb size (LMM: *F*_1,226.7_ = 1.04, *p *= .308, estimate ± *SE*: −0.74 ± 0.73, Table 3).

**Table 3:** Linear mixed‐effect models (LMMs) results for the relationship between social status and multiple behaviours on changes in body mass and comb size in female red junglefowl (*Gallus gallus*)

Full model	*n*	Fixed effects	Estimates	*SE*	*F*	ddf	*p*	σ^2^	τ00
BMc ~ BM	220	Intercept	58.36	30.32	–	70.85	–	2866.2	244.65
		Body mass	−0.06	0.03	3.78	69.45	.056		
BMc ~ Elo	200	Intercept	4.51	12.73	–	207.18	–	2920.81	173.09
		Elo score	<−0.01	<0.01	0.1	198.49	.751		
BMc ~ CC	200	Intercept	11.71	7.09	–	52.89	–	2946.97	114.01
		Coerced copulations	−0.29	0.14	4.31	152.25	**.039**		
BMc ~ TC	200	Intercept	9.79	8.21	–	55.53	–	2967.88	129.35
		Total copulations	−0.13	0.09	1.96	118.92	.164		
BMc ~ Co	200	Intercept	9.18	7.92	–	72.71	–	2937.58	175.25
		Courtship	−0.12	0.09	1.99	202.9	.159		
BMc ~ F	200	Intercept	7.19	7.68	–	72.64	–	2951.13	168.07
		Fecundity	−1.91	1.66	1.32	211.48	.252		
BMc ~ MS	200	Intercept	15.66	10.99	–	104.38	–	2953.96	140.63
		Mating success	−2.76	1.79	2.39	170.58	.124		
CSc ~ CS	235	Intercept	5.15	4.63	–	34.12	–	540.72	120.4
		Comb size	−0.03	0.01	4.18	218.43	**.042**		
CSc ~ CS* + Elo	235	Intercept	3.78	6	–	78.82	–	537.23	123.56
		Comb size	−0.03	0.01	4.02	216.06	**.046**		
		Elo score	<0.01	<0.01	0.21	212.35	.644		
CSc ~ CS* + CC	235	Intercept	10.1	5.26	–	55.27	–	541.19	101.32
		Comb size	−0.03	0.01	4.53	217.38	**.035**		
		Coerced copulations	−0.11	0.06	3.44	225.32	.065		
CSc ~ CS* + TC	235	Intercept	11.54	5.8	–	65.83	–	541.54	102.74
		Comb size	−0.03	0.01	5.04	217.59	**.026**		
		Total copulations	−0.08	0.04	3.24	207.52	.073		
CSc ~ CS* + Co	235	Intercept	5.2	5.29	–	48.07	–	543.07	120.89
		Comb size	−0.03	0.01	4.13	218.38	**.044**		
		Courtship	<−0.01	0.04	<0.01	231.72	.982		
CSc ~ CS* + F	235	Intercept	8.05	5.41	–	54.8	–	542.61	114
		Comb size	−0.03	0.01	4.37	216.92	**.038**		
		Fecundity	−0.74	0.73	1.04	226.73	.308		
CSc ~ CS* + MS	235	Intercept	9.67	6.4	–	88.53	–	543.27	112.48
		Comb size	−0.03	0.01	4.22	217.4	**.041**		
		Mating success	−0.81	0.79	1.06	213.04	.305		

Note

*p* values of fixed effects are based on *F* tests with Satterthwaite's approximation and are highlighted in bold when results are statistically significant (*p *< .05). For more detailed information on predictors, see methods.

Abbreviations: *, covariates; BM, body mass; BMc, body mass change; CC, number of coerced copulations; Co, courtship (i.e. number of waltzes received); CS, comb size; CSc, comb size change; Elo, Elo score; F, fecundity; MS, mating success; TC, total copulations; n, sample size; *SE*, standard error; *F*,* F* statistics; ddf, denominator degrees of freedom; σ^2^, residual variance of the random effects; τ00, sum of the random intercept variances of female identity, female group identity and group identity.

## 4 DISCUSSION

Investigating inter‐ and intraindividual patterns of variation in phenotypes associated with reproduction is critical to further our understanding of sex‐specific reproductive costs, sex‐specific selection, sexual conflict over reproductive decisions, condition dependence and the significance of ornaments and ornament preference. In the present study, we used replicate groups of red junglefowl to explore how inter‐ and intraindividual variation in body mass and comb size are related to social status and different behaviours related to mating effort in both males and females. First, we showed that body mass, but not comb size, was associated with social status across males, while comb size, but not body mass, was associated with social status across females. Second, over the course of a trial, both individual males and females on average tended to lose body mass, likely reflecting costs associated with activities related to exposure to the opposite sex, for example mating and intrasexual competition. Importantly, subordinate males lost more body mass than more dominant males, and this was likely due, at least in part, to subordinate males being chased more often by other males. In addition, males that mated more frequently and females that suffered more coerced copulations lost more body mass. Over the course of a trial, combs also became smaller on average in both individual males and females, suggesting that relative comb size reflects these general costs. However, we found no strong evidence that comb size changes within individuals were associated with individual social status or mating effort in either sex.

We show that, for a given comb size, body mass is positively associated with social status across males. This relationship may in part capture an effect of body size, because variation in the first measurement of body weight will reflect both skeletal size and residual body mass. Larger males may have a competitive advantage in social contests and thus be more likely to attain high rank in the hierarchy in fowl populations (Cornwallis & Birkhead, 2008; Graves et al., 1985; Johnsen et al., 2001; Ligon et al., 1990; Wilson et al., 2009). Conversely, we show that, for a given body mass, comb size is not statistically associated with male social status. This finding is inconsistent with some previous studies that have indicated a relationship between male comb size and social competitive ability (e.g. Graves et al., 1985; Johnsen et al., 2001; Ligon et al., 1990). For example, Ligon et al. (1990) showed that large‐combed males tend to be more aggressive and therefore more likely to initiate and win fights. It is however difficult to assess the independent role of comb size given that males with large combs are often heavier (Cornwallis & Birkhead, 2008; Graves et al., 1985; Johnsen et al., 2001; Ligon et al., 1990; this study). Here, we attempted to overcome this difficulty by controlling statistically for the effect of body mass when we investigated the association between comb size and social status. Moreover, several studies have used pairs of males with pre‐selected differences in comb size, whereas here, comb size and status were analysed under arguably more natural conditions, as continuous variables within larger groups of freely‐interacting males. Our results are consistent with the idea that intrasexual selection favours larger, heavier males, but do not support the idea that independent variation in comb size is informative of male social status. However, comb size may covary with male status only to the extent to which comb size is associated with male body size/mass. Finally, our finding that male comb is an unreliable indicator of social status complements recent findings in other species challenging the idea that male ornaments may function as ‘badges of status’ (Sánchez‐Tójar, Nakagawa, et al., 2018).

These results have implications for patterns of intra‐ *versus* intersexual episodes of sexual selection, given that females have been shown to prefer males with large combs (Ligon & Zwartjes, 1995, 1995; Zuk, Johnson, Thornhill, Ligon, & David, 1990; Zuk, Thornhill, Ligon, Johnson, et al., 1990; Zuk et al., 1995). Our results call into question the adaptive significance of female preference for male comb size since, in addition to the lack of association between comb size and social status as described above, we found little evidence that male comb size is particularly sensitive to costs associated with mating effort and intrasexual competition. Although males generally experienced comb shrinking over the course of a trial, suggesting that relative comb size can reflect general costs associated with exposure to females, we failed to find consistent changes in comb size in relation to male status or mating effort, suggesting that relative changes within males are unlikely to inform females of male status or condition. An important caveat is that status‐dependent comb size differences may require a longer period of time to emerge, for example through the reinforcement of hierarchical relationships between males, than that allowed in our study. This does not appear likely however, given that birds were given 3 days to establish sex‐specific hierarchies prior exposure to the opposite sex, and the study detected status‐dependent variation in body mass. The lack of a relationship between male comb size and social status has implications for the maintenance of variation. If large‐combed males were also dominant on average, female choice would align with, and reinforce, intrasexual selection on dominance and its covariate body mass (Hunt, Breuker, Sadowski, & Moore, 2009). On the other hand, a lack of positive covariance between social status and comb size combined with female preference for large‐combed males would contribute to the maintenance of variation. In addition, the extent to which male status and comb size influence female preference may be socially modulated. For example, Johnsen et al. (2001) showed that, in small red junglefowl flocks comprising three females and two males, top‐ and second‐ranking females consistently mated with the dominant male independently of his comb size, while bottom‐ranking females tended to mate with the subordinate male if he had a larger comb. However, female preference for large combs may not be actualized if female access to preferred males is curtailed by dominant males (Cheng & Burns, 1988; Dean, Nakagawa, & Pizzari, 2011; Pizzari, 2001).

As with males, female comb tended to shrink over the course of the trial, but we did not find any consistent association between these changes and the female status or mating frequency. However, we did find that across individuals, dominant females tended to have a larger comb, which is in line with the majority of previous studies (e.g. Bradshaw, 1992; Collias, 1943; Forkman & Haskell, 2004; Guhl & Ortman, 1953; Martin et al., 1997) with few exceptions. Studying an intercross of domestic strains, Cloutier and Newberry (2000) found that female status was associated with comb size in one of two trials and with body weight across both trials. Similarly, Zuk et al. (1998) found that in red junglefowl groups, female social status was associated with body weight but not comb size. Differences in the relationship between social status and comb size in females *versus* males in the present study could reflect the social circumstances under which social status was measured in different sexes (e.g. Procter, Moore, & Miller, 2012). While male social hierarchies were based on interactions in mixed‐sex groups, female social hierarchies were assessed in single sex groups. More generally, the relationship between female status and comb size likely emerges as a function of female aggressiveness, which, together with comb size, is modulated by circulating levels of steroid hormones (Allee et al., 1939; Collias, 1943; Guhl & Ortman, 1953). Dominant females initiate most of the aggressive interactions with their peers (Kim & Zuk, 2000), and females injected with testosterone climbed their way up the pecking order when reintroduced into their previous flock (Allee et al., 1939), whereas the opposite occurred with females that had their combs surgically removed (Marks, Siegel, & Kramer, 1960). This is interesting since, while female ornamentation is often explained as simply the result of a genetic correlation with male ornamentation (Lande, 1980), recent studies indicated that ornaments may fulfil adaptive functions in females (Amundsen, 2000). For example, female ornaments may increase mate acquisition by signalling fecundity (Domb & Pagel, 2001; Potti, Canal, & Serrano, 2013) or may evolve as a function of female competition for resources (e.g. Heinsohn, Legge, & Endler, 2005). In the fowl, female comb size has been positively associated with egg investment (Cornwallis & Birkhead, 2007; Pizzari et al., 2003; Wright et al., 2008), and evidence suggests that dominant males prefer females with larger combs (Cornwallis & Birkhead, 2007; Pizzari et al., 2003). Therefore, the covariance between status and comb size suggests that large‐combed, dominant females may have preferential access to resources and mating opportunities, likely underpinning the positive female Bateman's gradient described for this species (Collet et al., 2014). Supporting this possibility, Collias et al. (1994) and recent work with this population (McDonald et al., in press) have shown that, controlling for age, dominant females tend to have higher reproductive success. The negative quadratic relationship between female comb size and status found in our study is similar to curvilineal relationships between female comb size and both egg number and weight found by Wright et al. (2008). These patterns suggest that, beyond a certain size, comb size is no longer informative of female status and egg investment.

Both sexes tended to lose body mass during the course of a trial. Similarly, Zuk and Johnsen (2000) found that both subordinate and dominant males lost body mass after being moved from male‐only pens to experimental pens with females. In both Zuk and Johnsen (2000) and our present study, birds had *ad libitum* food and water, and the main difference from their pre‐experiment housing was larger group size and exposure to the opposite sex, suggesting that the overall loss in body mass was at least partially driven by activities associated with courting, mating effort and intrasexual competition over access of mating opportunities. Supporting this conclusion, we show that, when holding status constant, males that mated more often tended lose more mass, highlighting a possible cost of mating. This cost may have been exacerbated in our study due to the polyandry of these groups, demanding males to re‐mate often with a female to defend paternity in sperm competition (McDonald et al., 2017). An increase in copulation frequency in the presence of competitors has been described in other species of birds (e.g. Crowe et al., 2009; Sax, Hoi, & Birkhead, 1998). While studies have typically focused on cost of mating to females (Daly, 1978), there is increasing evidence that exposure to females and mating can exact substantial costs on males. Costs associated with courting females and mating have been documented in several invertebrate species (Cordts & Partridge, 1996; Kotiaho, 2000; Mappes, Alatalo, Kotiaho, & Parri, 1996; Paukku & Kotiaho, 2005) and in some vertebrates. For example, in insectivorous marsupials, polyandry and a brief mating season select for male investment in testis size and longer copulations, which leads to immune collapse followed by death (Fisher, Dickman, Jones, & Blomberg, 2013). In a less extreme example, Preston, Stevenson, Pemberton, and Wilson (2001) showed that dominant male Soay sheep (*Ovis aries*) which copulate frequently and earlier in the breeding season, benefit from early reproductive success, but suffer a decline in reproductive performance due to sperm depletion later on the breeding season. Similarly, a recent study with mosquitofish (*Gambusia holbrooki*) showed that males raised with females grew slower and showed reduced immune response, compared to sexually naïve males, highlighting possible costs of mating (Iglesias‐Carrasco, Fox, Vincent, Head, & Jennions, 2019).

Independent of mating costs, we also find evidence of a cost of social competition in males. Both tenure of high and low social rank have been suggested to be associated with substantial energetic and physiological costs. Here, we show that in red junglefowl groups, subordinate males tended to lose more body mass than dominant males, suggesting that subordination is relatively costly. In accordance with this idea, we were able to show that the degree of body mass loss in a male was predicted by the frequency at which he was chased by other males. In fowl, chasing subordinate males, which is sometimes followed by physical aggression, is a frequent behaviour that dominant males use to interrupt copulation attempts from subordinates and reinforce their dominance status (Cheng & Burns, 1988; Dean et al., 2011; Pizzari, 2001). The loss in body mass associated with subordinate status and the frequency at which subordinates were chased are in agreement with a vast body of literature that has found an association between stressing factors and subordination in captive populations (Abbott et al., 2003; Creel, 2001; Goymann & Wingfield, 2004). For example, a decrease in body mass is a frequently described effect of social stress in subordinate laboratory rats (reviewed in Tamashiro et al., 2007). Alternatively, high and low rank may be associated with similar costs but dominant males find these costs easier to bear. This explanation would be consistent with our finding that heavier males are more likely to achieve a higher rank and could in part be mediated by the privileged access of dominant males to resources such as food, perching and dustbathing spots (Pizzari & McDonald, 2019).

Finally, our study presents new evidence that male sexual coercion may lead to costs to females, as shown by the increased loss in body mass in females that experience frequent male harassment. However, because sexual harassment is conflated with the act of mating, we cannot completely rule out a contribution of mating *per se* to losses in body mass. We find this to be unlikely however, since the total number of copulation attempts was not statistically associated with changes in female body mass. More generally, we show that these costs to females are comparable to the rate of body mass loss suffered by males. For example, the body mass reduction on average associated with each *SD* increment in male mating success (11.91 g) represents approximately 0.85% of a male body mass (average 1392.55 g). Similarly, *SD* increments in copulation attempts or being chased by other males correspond on average to 0.61% and 0.59% of average male body mass, respectively. In comparison, we show that one *SD* increment in sexual harassment suffered by females corresponds on average to approximately 0.77% loss of female body mass (average 1035.29 g). The energetic cost of male harassment is likely one of the selective pressures leading to female strategies of male avoidance (Løvlie & Pizzari, 2007; Pizzari, 2001). This pattern of sexual conflict is expected when intrasexual competition for mating opportunities is intense among males (Chapman, Arnqvist, Bangham, & Rowe, 2003; Parker, 2006). Female costs associated with mating, sexual coercion and male harassment have been well documented (Arnqvist & Rowe, 2005). This evidence, however, is often restricted to laboratory‐adapted invertebrate populations, and female costs of mating are rarely empirically quantified in more natural populations, although they are likely substantial. For example, female water striders (*Aquarius remigis*) struggling to reject attempted copulations from males can spend approximately 200% more energy carrying the harassing male on their back, compared with normal locomotion (Watson, Arnqvist, & Stallmann, 1998). Extreme cases where females are injured or even killed by males have been documented in several vertebrates (e.g. Chilvers, Robertson, Wilkinson, Duignan, & Gemmell, 2005; Le Galliard, Fitze, Ferriere, & Clobert, 2005; Réale, Boussès, & Chapuis, 1996). In our study, females could have lost body mass due to the energetic expenditure of struggling against males and/or due to high stress levels as a consequence of persistent harassment. Alternatively, the loss in body mass could have been a consequence of male disruption of feeding behaviour. For example, male sexual harassment substantially decreases female foraging time in both guppies (*Poecilia reticulata*) and Atlantic mollies (*P. mexicana*) (Magurran & Seghers, 1994; Plath, Parzefall, & Schlupp, 2003), whereas females of the solitary bee *Anthophora plumipes* are forced to feed in less rewarding parts of the flower to escape male harassment (Stone, Road, & Ox, 1995). These results suggest a striking potential for the structure of social groups to influence the condition and productivity of groups through costs imposed to females. By reducing female condition, male harassment is likely to increase the male bias of the operational sex ratio of the population, thus exacerbating the intensity of sexual harassment suffered by the few females that are reproductively available, triggering a negative feedback loop which may lead to local population extinction (Le Galliard et al., 2005).

In conclusion, our study helps resolve the relationship between a fleshy ornament (comb size) and social status in fowl. We demonstrate that body mass, rather than comb size, is consistently associated with status in males, which suggests that female choice and male–male competition target different male traits. Importantly, we show that body mass changes dynamically over time in response to social status and mating effort in males and in response to male sexual harassment in females. These results highlight the sex‐specific costs of mating and their implications for temporal changes in an individual's phenotype over a mating season. Our results also show how such dynamics can have important ramifications for the maintenance of variation in sexually selected traits. Future studies should explore the way in which these patterns are modulated by factors such as seasonality, group size and sex ratio. This work may also inform better management of productivity and animal welfare in commercial flocks and captive populations (Pizzari, 2016).

## DATA ARCHIVING

Data archival statement: Data supporting this article are available on DRYAD (https://doi.org/10.5061/dryad.tm8038t).
