# Supplementary material for: Dynamic phenotypic correlates of social status and mating effort in male and female red junglefowl, Gallus gallus
Source: J Evol Biol. 2019 Sep 28;33(1):22–40. doi: 10.1111/jeb.13541 (PMC6972591; doi:10.1111/jeb.13541)
Supplement: Supplementary file 1 [file JEB-33-22-s001.docx]

**Online supplementary materials for: Dynamic phenotypic correlates of social status and mating effort in male and female red junglefowl, *Gallus gallus***

**Supplementary table:**

**Supplementary figures:**


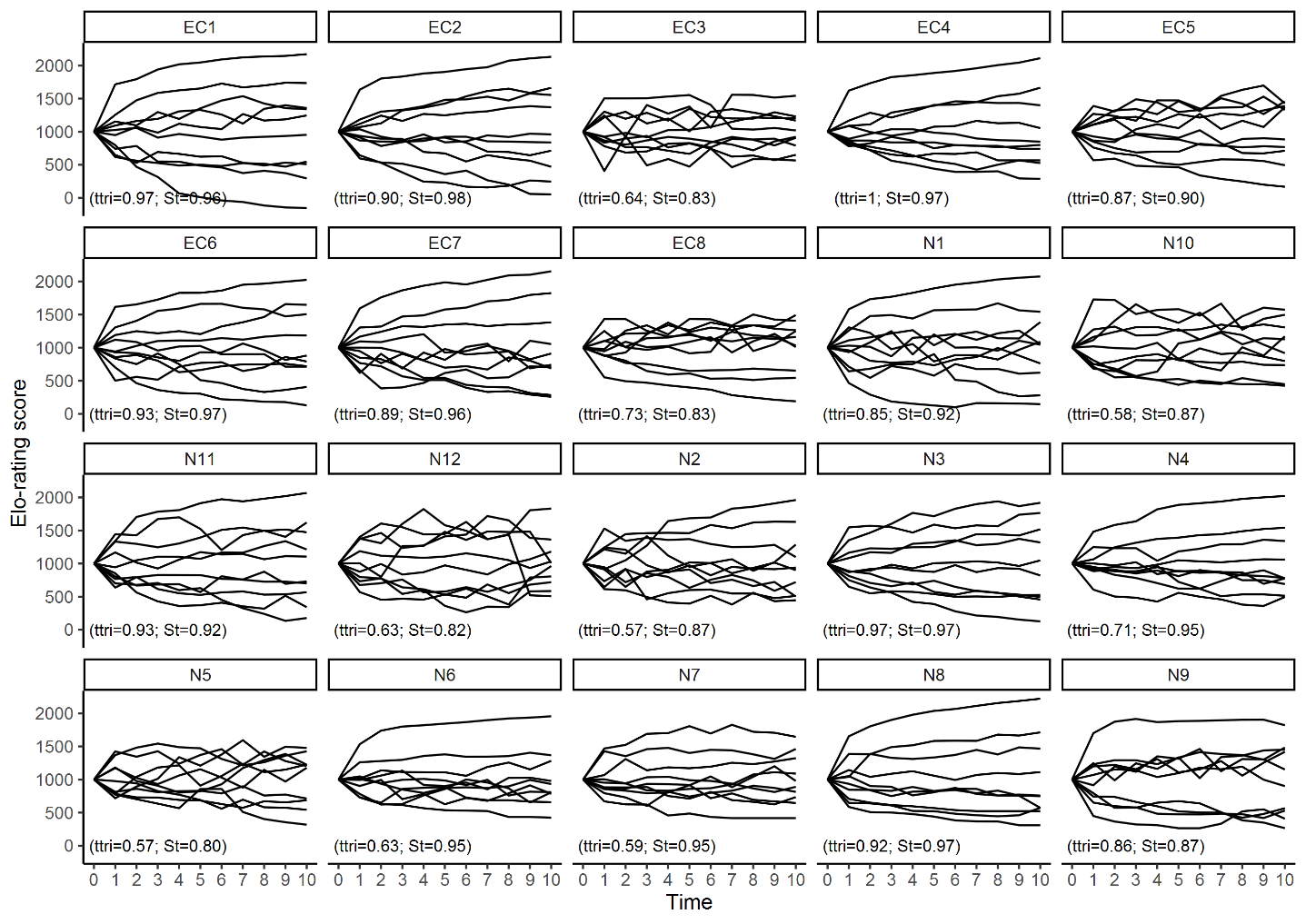


Figure S1 – Trajectories of males’ Elo-rating scores over the 10 days of mixed-sex trials. Each panel represents one of the 20 replicate groups, with each individual line representing a male red junglefowl (*Gallus gallus*). In parenthesis we provide the dominance hierarchy transitivity (t_tri_) and stability (S_t_). The former measures the proportion of transitive triads in the hierarchy, and the latter measures how often males swap ranks. Values range from 0 (less transitive or stable) to 1 (more transitive or stable).


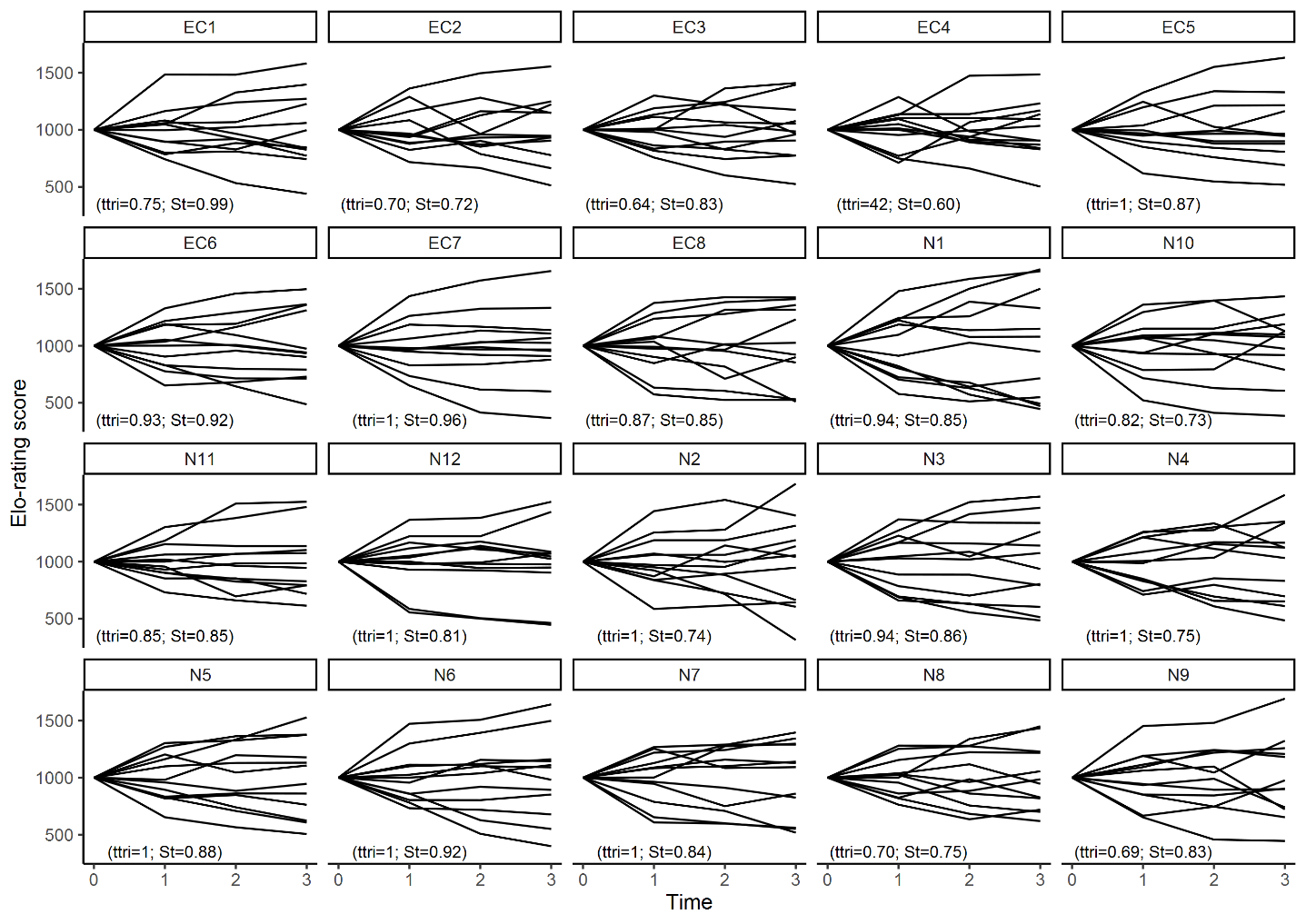


Figure S2 – Trajectories of females’ Elo-rating scores over the three days of female-only trials. Each panel represents one of the groups of 12 females used in each mixed-sex trial, with each individual line representing a female red junglefowl (*Gallus gallus*). In parenthesis we provide the dominance hierarchy transitivity (t_tri_) and stability (S_t_). See figure S1.


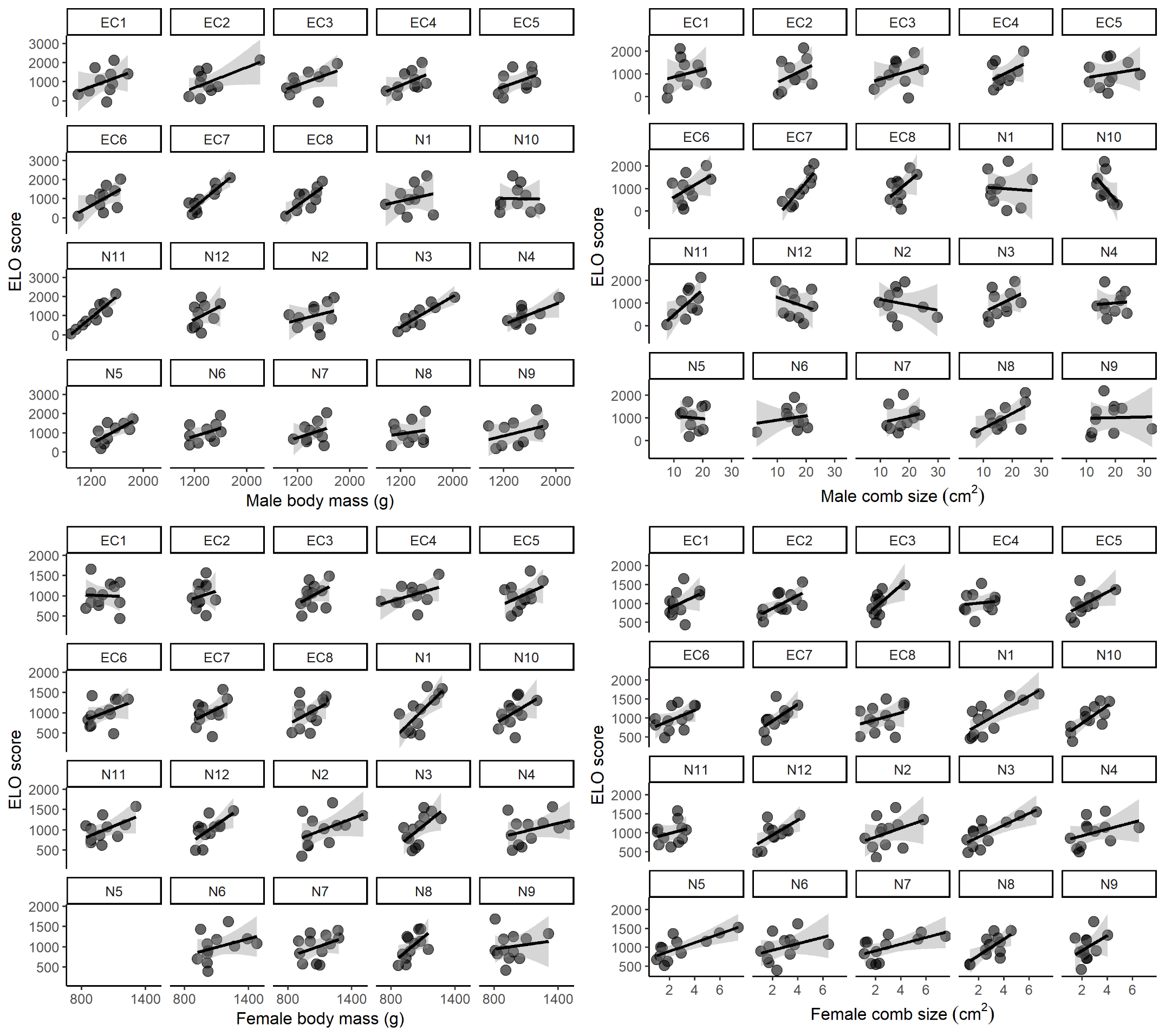


Figure S3 – Relationship between body mass or comb size and social status across the 20 mixed-sex replicate groups of red junglefowl (*Gallus gallus*). Males are represented on the top two graphs and females at the bottom. Data points represent individual birds, with darker regions indicating data point overlaps. Shaded areas around the regression line represent the 95% confidence intervals. We lacked information on female body mass for group N5.


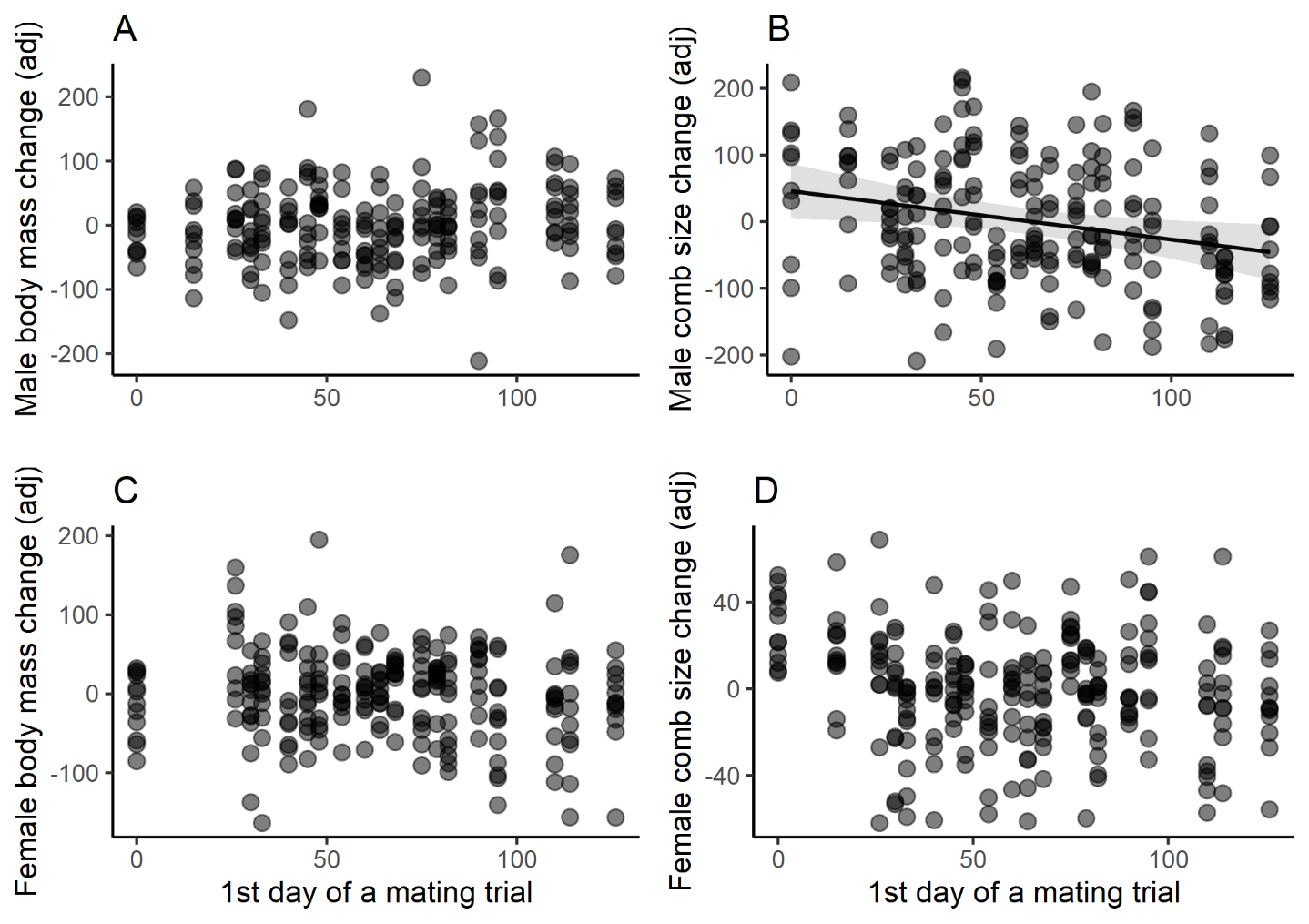


Figure S4 – Variation in body mass and comb size change in relation to seasonality in male and female red junglefowl (*Gallus gallus*). Changes in body mass over the breading season in males (A) and females (C); Changes in comb size over the breading season in males (B) and females (D). Data points represent individual birds, with darker regions indicating data point overlaps. Shaded areas around the regression line represent the 95% confidence intervals.

#######R SCRIPT FOR MAIN FINDINGS IN “Dynamic phenotypic correlates of social

#######status and mating effort in male and female red junglefowl, *Gallus gallus”*

# Clear directory

rm(list=ls())

# load required packages

library(sjPlot)

library(lme4)

library(ggplot2)

library(dplyr)

library(lmerTest)

library(car)

full_male # Male dataset

full_female # Female dataset

###Relationship between male status, body mass comb size and age

mod1<- lmer(meanELO_SCORE ~ Body_Mass_Before + Comb_Area_Before + Age +

(1|MaleID) + (1|femalegroupID) + (1|groupID), full_male)

plot(mod1) # Check homoscedasticity

qqnorm(resid(mod1)) # Check for non-normality of the residuals

qqline(resid(mod1))

vif(mod1) # Check for collinearity of the predictors

Body_Mass_Before Comb_Area_Before Age

1.761621 1.409007 1.484716

anova(mod1) # Hypothesis testing

Type III Analysis of Variance Table with Satterthwaite's method

Sum Sq Mean Sq NumDF DenDF F value Pr(>F)

Body_Mass_Before 6799558 6799558 1 133.50 29.9377 2.13e-07 ***

Comb_Area_Before 3568 3568 1 124.72 0.0157 0.9005

Age 1344 1344 1 118.09 0.0059 0.9388

---

Signif. codes: 0 ‘***’ 0.001 ‘**’ 0.01 ‘*’ 0.05 ‘.’ 0.1 ‘ ’ 1

## Plot the marginal effect of body mass on Elo score

plot_model(mod1, terms = "Body_Mass_Before”, type = "pred",

show.data = T)

###Relationship between female status, body mass comb size,

###age and fecundity

mod2 <- lmer(meanELO_SCORE ~ Comb_Area_Before + I(Comb_Area_Before^2)

+ Age + N_eggs + Body_Mass_Before + (1 | groupID) +

(1 | FemaleID) + (1|femalegroupID), full_female)

plot(mod2) # Check homoscedasticity

qqnorm(resid(mod2)) # Check for non-normality of the residuals

qqline(resid(mod2))

vif(mod2) # Check for collinearity

Comb_Area_Before I(Comb_Area_Before^2) Age

11.489889 11.445135 1.294611

N_eggs Body_Mass_Before

1.021512 1.429766 # CS and (CS)^2^ are collinear,

# but this was expected

anova(mod2)

Type III Analysis of Variance Table with Satterthwaite's method

Sum Sq Mean Sq NumDF DenDF F value Pr(>F)

Comb_Area_Before 232848 232848 1 143.289 10.2343 0.001697 **

I(Comb_Area_Before^2) 90490 90490 1 137.300 3.9773 0.048097 *

Age 739829 739829 1 79.606 32.5177 1.932e-07 ***

N_eggs 134106 134106 1 207.915 5.8944 0.016041 *

Body_Mass_Before 385 385 1 187.406 0.0169 0.896645

---

Signif. codes: 0 ‘***’ 0.001 ‘**’ 0.01 ‘*’ 0.05 ‘.’ 0.1 ‘ ’ 1

# Plot the marginal effect of comb size on elo score

plot_model(mod2, terms = "Comb_Area_Before[all]",

type = "pred", show.data = T)

###Association between male status and changes in body mass

mod3<- lmer(Corrected_mass ~ Body_Mass_Before + meanELO_SCORE +

(1|MaleID) + (1|femalegroupID) +

(1|groupID), full_male)

plot(mod3) # Check homoscedasticity

qqnorm(resid(mod3)) # Check for non-normality of the residuals

qqline(resid(mod3))

vif(mod3) # Check for collinearity

Body_Mass_Before meanELO_SCORE

1.269319 1.269319

anova(mod3)

Type III Analysis of Variance Table with Satterthwaite's method

Sum Sq Mean Sq NumDF DenDF F value Pr(>F)

Body_Mass_Before 144650 144650 1 153.22 60.558 9.869e-13 ***

meanELO_SCORE 51391 51391 1 187.27 21.515 6.578e-06 ***

---

Signif. codes: 0 ‘***’ 0.001 ‘**’ 0.01 ‘*’ 0.05 ‘.’ 0.1 ‘ ’ 1

###Association between times being chased and changes in body mass,

###controlling for social status and initial body mass in males

mod4<- lmer(Corrected_mass ~ Body_Mass_Before + meanELO_SCORE

+ Chased + (1|MaleID) + (1|femalegroupID) +

(1|groupID), full_male)

plot(mod4) # Check homoscedasticity

qqnorm(resid(mod4)) # Check for non-normality of the residuals

qqline(resid(mod4))

vif(mod4)

Body_Mass_Before meanELO_SCORE Chased

1.307959 1.527056 1.205271

anova(mod4)

Type III Analysis of Variance Table with Satterthwaite's method

Sum Sq Mean Sq NumDF DenDF F value Pr(>F)

Body_Mass_Before 127182 127182 1 154.84 54.5205 8.871e-12 ***

meanELO_SCORE 27677 27677 1 195.72 11.8648 0.0006998 ***

Chased 9372 9372 1 136.21 4.0177 0.0470072 *

---

Signif. codes: 0 ‘***’ 0.001 ‘**’ 0.01 ‘*’ 0.05 ‘.’ 0.1 ‘ ’ 1

###Association between mating success and changes in body mass,

###controlling for social status and initial body mass in males

mod5<- lmer(Corrected_mass ~ Body_Mass_Before + meanELO_SCORE +

Mating_success + (1|MaleID) + (1|femalegroupID) +

(1|groupID), full_male)

plot(mod5) # Check homoscedasticity

qqnorm(resid(mod5)) # Check for non-normality of the residuals

qqline(resid(mod5))

vif(mod5)

Body_Mass_Before meanELO_SCORE Mating_success

1.276993 1.313250 1.039250

anova(mod5)

Type III Analysis of Variance Table with Satterthwaite's method

Sum Sq Mean Sq NumDF DenDF F value Pr(>F)

Body_Mass_Before 149756 149756 1 159.91 68.1337 5.384e-14 ***

meanELO_SCORE 61390 61390 1 189.07 27.9302 3.446e-07 ***

Mating_success 20651 20651 1 177.77 9.3954 0.002515 **

---

Signif. codes: 0 ‘***’ 0.001 ‘**’ 0.01 ‘*’ 0.05 ‘.’ 0.1 ‘ ’ 1

###Association between number of coerced copulation and changes in body mass,

###controlling for social status and initial body mass in females

mod6 <- lmer(Corrected_mass ~ Resisted_cop + (1 | groupID)

+ (1 | FemaleID) + (1|femalegroupID), full_female)

plot(mod5) # Check homoscedasticity

qqnorm(resid(mod5)) # Check for non-normality of the residuals

qqline(resid(mod5))

anova(mod6)

Type III Analysis of Variance Table with Satterthwaite's method

Sum Sq Mean Sq NumDF DenDF F value Pr(>F)

Resisted_cop 12691 12691 1 152.25 4.3064 0.03965 *

---

Signif. codes: 0 ‘***’ 0.001 ‘**’ 0.01 ‘*’ 0.05 ‘.’ 0.1 ‘ ’
